# Pomalidomide Plus Low-Dose Dexamethasone in Relapsed/Refractory Multiple Myeloma Patients: Results of the Real-World “POWERFUL” Study

**DOI:** 10.3390/jcm10071509

**Published:** 2021-04-05

**Authors:** Evangelos Terpos, Panagiotis Repousis, Chrysavgi Lalayanni, Evdoxia Hatjiharissi, Theodora Assimakopoulou, Georgios Vassilopoulos, Anastasia Pouli, Emmanouil Spanoudakis, Eurydiki Michalis, Gerassimos Pangalis, Ioannis Ntanasis-Stathopoulos, Christos Poziopoulos, Marie-Christine Kyrtsonis, Vasiliki Pappa, Argiris Symeonidis, Christos Georgopoulos, Panagiotis M. Zikos, Maria Gavriatopoulou, Helen A. Papadaki, Magdalini Dadakaridou, Kiki Karvounis-Marolachakis, Eirini Katodritou

**Affiliations:** 1 Department of Clinical Therapeutics, School of Medicine, National and Kapodistrian University of Athens, Alexandra General Hospital, 11527 Athens, Greece; eterpos@med.uoa.gr (E.T.); johnntanasis@med.uoa.gr (I.N.-S.); mariagabria@gmail.com (M.G.); 2 Department of Hematology, “METAXA” Cancer Hospital of Piraeus, 18537 Athens, Greece; repousis@otenet.gr (P.R.); mag.dadak@gmail.com (M.D.); 3 Department of Hematology, «G. PAPANIKOLAOU» General Hospital of Thessaloniki, 57010 Thessaloniki, Greece; luizana6@gmail.com; 4 1st Department of Internal Medicine, Division of Hematology, AHEPA University General Hospital of Thessaloniki, 54636 Thessaloniki, Greece; ehatjiharissi@gmail.com; 5 Department of Hematology, “SISMANOGLIO-AMALIA FLEMING” General Hospital of Athens, Marousi, 15126 Athens, Greece; theass56@yahoo.gr; 6 Department of Hematology, Larissa University Hospital, 41110 Larissa, Greece; gvasilop@icloud.com; 7 Department of Hematology, “AGIOS SAVVAS” Anticancer-Oncology Hospital of Athens, 11522 Athens, Greece; a.pouli@hotmail.gr; 8 Department of Hematology, University Hospital of Alexandroupolis, 68100 Alexandroupolis, Greece; emmanouilspanoudakis@yahoo.com; 9 Department of Hematology, “G. GENNIMATAS” General Hospital of Athens, 11527 Athens, Greece; eviamich@gmail.com; 10 Department of Hematology, Athens Medical Center-Psychikon Branch, Psychiko, 11525 Athens, Greece; pangalis@med.uoa.gr; 11 Department of Hematology, “METROPOLITAN” Athens Private Hospital, N. Faliro, 18547 Athens, Greece; cpozi@otenet.gr; 12 First Department of Propaedeutic Internal Medicine, Laikon General Hospital, National and Kapodistrian University of Athens, 11527 Athens, Greece; mckyrtsonis@gmail.com; 13 2nd Department of Internal Medicine and Research Unit, Hematology Unit, “ATTIKON” University General Hospital of Athens, School of Medicine, National and Kapodistrian University of Athens, Haidari, 12462 Athens, Greece; vas_pappa@yahoo.com; 14 Department of Hematology, University General Hospital of Patras, Rio, 26504 Patras, Greece; argiris.symeonidis@yahoo.gr; 15 Department of Hematology, 424 General Military Hospital of Thessaloniki, 56429 Thessaloniki, Greece; ch.georgo64@gmail.com; 16 Department of Hematology, “Agios Andreas” General Hospital of Patras, 26335 Patras, Greece; pmzikos@gmail.com; 17 Department of Hematology, University General Hospital of Heraklion, School of Medicine, University of Crete, 71110 Heraklion, Greece; e.papadaki@uoc.gr; 18 Medical Department, Genesis pharma SA, Halandri, 15232 Athens, Greece; kkarvouni@genesispharma.com; 19 Department of Hematology, “THEAGENIO” Anti-Cancer Hospital of Thessaloniki, 54007 Thessaloniki, Greece

**Keywords:** duration of response, lenalidomide, multiple myeloma, pomalidomide, PFS, ORR, refractory

## Abstract

The “POWERFUL” multicenter, retrospective, and prospective study investigated the effectiveness of pomalidomide plus low-dose dexamethasone (POM/LoDex) therapy in relapsed/refractory multiple myeloma in routine care in Greece. Ninety-nine eligible adult patients treated with POM/LoDex according to the approved label after having received ≥2 prior therapies, including lenalidomide and bortezomib, were consecutively enrolled between 16 November 2017 and 21 February 2019 in 18 hematology departments. Fifty patients (50.5%) started POM/LoDex as third-line treatment. During the treatment period (median: 8.3 months; range: 0.3–47.6 months), the median POM dose was 4 mg/day, and 31.3% of the patients received additional antimyeloma agents. The overall response rate was 32.3%. During a median follow-up period of 13.8 months (Kaplan–Meier estimate), the median progression-free survival (PFS) was 10.5 months (95% CI: 7.4–14.4). The PFS was not significantly different between patients receiving POM/LoDex in the third versus later line of therapy, nor between patients receiving concomitant antimyeloma therapy versus POM/LoDEx doublet. During the prospective safety data collection period (median: 7.6 months) among patients with prospective follow-up (N = 75), POM-related adverse event incidence rate was 42.7% (serious: 18.7%; grade  ≥  3 hematological POM-related adverse events: 8.0%). Only neutropenia (13.3%) was reported at a frequency ≥10%. In conclusion, in this real-world study, POM/LoDex displayed a long PFS with no new safety signals emerging.

## 1. Introduction

Multiple myeloma (MM), a hematological malignancy associated with significant morbidity and mortality, is characterized by clonal plasma cell expansion in the bone marrow, presence of monoclonal immunoglobulin in the serum or urine, lytic bone lesions, hypercalcemia, renal insufficiency, anemia, and immunodeficiency. Currently, in addition to conventional chemotherapy and autologous stem cell transplantation (ASCT), treatment options for MM include immunomodulatory agents (IMiDs), proteasome inhibitors (PIs), monoclonal antibodies, and histone deacetylase inhibitors, as well as the more recently introduced first generation Xportin-1 inhibitor, selinexor [1,2,3,4]. Despite advances in the field, therapeutic management of MM remains challenging, as patients invariably relapse, develop drug resistance, and become refractory to treatment. As the disease progresses, genetic heterogeneity and immunosuppression increase, leading to shorter duration of response (DoR) and negatively affecting patients’ overall survival (OS) [5,6]. Therefore, effective treatment of early relapses with therapeutic approaches that not only provide direct tumoricidal effects but also suppress residual disease via immune mechanisms of action is critical in order to delay the onset of subsequent relapses [7,8].

Pomalidomide (POM), along with thalidomide and lenalidomide (LEN), belongs to the class of IMiDs. The drug has pleiotropic functions, via induction of myeloma cell apoptosis, inhibition of angiogenesis, and immunomodulation, and it targets not only tumor cells, but also the bone marrow microenvironment [9]. At the molecular level, all three IMiDs exert their activity by binding to cereblon (CRBN), a protein component of the CRL4 E3 ligase complex [10], inducing its interaction with the transcriptional factors Aiolos and Ikaros [11]. Ubiquitination and subsequent degradation of the two factors result in changes in the expression of a number of downstream genes, including IRF4, interleukin 2, and MYC, leading to cell cycle arrest and induction of apoptosis, as well as modulation of the bone marrow microenvironment and enhancement of immune response [12,13]. Pomalidomide is more potent than LEN and thalidomide against CRBN and displays distinct immune modulating properties. In addition, POM is more effective than LEN in ubiquitinating ARID2, a substrate of CRBN CRL4 and a component of the polybromo-associated BAF chromatin-remodeling complex, leading to its degradation, which in turn inhibits MYC expression and proliferation [14]. Interestingly, POM has demonstrated activity in in vitro and in vivo models of LEN resistance [7]. 

In combination with low-dose dexamethasone (LoDex), POM has been approved for the treatment of patients with RRMM who have experienced disease progression after at least two prior therapies, including LEN and bortezomib. In the clinical trial setting, POM/LoDex conferred a clinical benefit in terms of progression-free survival (PFS), time to progression (TTP), OS, and overall response rate (ORR) [15,16]. Subsequent studies demonstrated that the reported effects are independent of the number of prior therapies and refractoriness to LEN and/or bortezomib, whereas the survival benefit is extended to patients with renal impairment and high-risk cytogenetics [17,18,19,20]. However, real-world evidence on the effectiveness of POM/LoDex in RRMM is scarce. 

In light of the above, the “POWERFUL” retrospective chart review and prospective observational study aimed to evaluate the effectiveness and utilization patterns of POM/LoDex treatment in routine clinical care setting in Greece. The results indicate that POM/LoDex is an effective treatment option, with a manageable safety profile, that can be used in LEN and bortezomib refractory patients and even immediately after LEN failure.

## 2. Materials and Methods

### 2.1. Study Design and Population

POWERFUL was a non-interventional, multicenter, retrospective chart review and prospective cohort study that included patients with RRMM who were initiated on POM/LoDex in the third or later treatment line setting under routine care conditions in Greece. The study was carried out by hospital-based hematology specialists in geographically diverse locations throughout Greece.

Patient enrollment started with a 15-month prospective recruitment phase, during which patients who had received up to one cycle of POM/LoDex were consecutively enrolled. This phase was complemented with a 1-month retrospective chart review phase, which was initiated 1 month before the end of the prospective phase in order to facilitate the recruitment of the study population. During the retrospective phase, patients on POM/LoDex for more than 1 cycle or those who had discontinued treatment with POM/LoDex were consecutively enrolled in reverse chronological order, on the basis of POM/LoDex start date. 

Patients on POM/LoDex at enrollment were prospectively observed until the completion of a 12-month on-study period of the last enrolled patient, or until disease progression (PD), death, withdrawal of consent, treatment discontinuation, initiation of other anti-myeloma therapy without documented disease progression, study completion, or physician’s decision, whichever occurred earlier. For patients enrolled during the retrospective recruitment phase who had discontinued POM/LoDex or were deceased, we abstracted data from medical charts from MM diagnosis up to the earliest time point of PD on POM/LoDex, death, permanent treatment discontinuation of POM/LoDex, or start of other anti-myeloma therapy in the absence of PD on POM/LoDex. 

Eligible patients were adults diagnosed with RRMM on the basis of the International Myeloma Working Group (IMWG) or the European Group for Blood and Marrow Transplantation criteria who were initiated on POM/LoDex between 1 January 2016 and 28 February 2019 according to the locally approved label. The prescription of POM was clearly separated from the physician’s decision to include the patient in the current study. All patients had received at least 2 prior therapies including both LEN and bortezomib and had experienced disease progression after the last treatment. Patients with prior malignancy (within the 3 years preceding initial diagnosis of MM), patients receiving anti-cancer regimens for malignancies other than MM, and patients participating or who had participated in any interventional investigation program during the treatment phase were excluded from the study.

This study was designed and conducted in accordance with the principles of the International Society for Pharmacoepidemiology guidelines for Good Pharmacoepidemiology Practice, the ethical principles laid down in the Declaration of Helsinki, the STROBE (Strengthening the Reporting of Observational Studies in Epidemiology) guidelines where applicable, and all applicable local rules and regulations. All patients provided a signed informed consent form, while a waiver of consent, provided by the Hospital Scientific Committee and/or Administrative Board of the study site, was required for retrospective enrollment of deceased individuals.

### 2.2. Study Objectives and Relevant Definitions 

The study’s primary objective was to evaluate the effectiveness of POM/LoDex in terms of median PFS, while secondary objectives included the evaluation of the 12-month PFS, the treatment response in terms of overall response rate (ORR, defined as achievement of at least a partial response (PR)), clinical benefit rate (CBR, defined as achievement of at least a minimal response (MR)), disease control rate (DCR, defined as achievement of at least stable disease), as well as the estimation of the time to response (TTR) and the DoR among patients who achieved at least PR. In addition, the study aimed to describe real-world utilization patterns of POM/LoDex in terms of the rate of its incorporation in the third versus later lines of therapy, prior antimyeloma therapies, POM starting dose, dose modifications, rate of temporary and permanent interruptions, and administration of prophylactic medications. Furthermore, the study aimed to examine the association between patient, disease, and treatment characteristics with the effectiveness of POM/LoDex. The evaluation of effectiveness of Pom/LoDex in patients with RRMM in the third versus later-line setting was an exploratory study objective.

### 2.3. Statistical Methods

All effectiveness analyses were performed in the overall eligible population. Safety analysis was performed in the safety evaluable population comprised of patients with prospective follow-up. According to the study protocol, adverse events (AEs) were collected and reported from informed consent until 28 days after the date of last dose of POM received during the study; no retrospective safety data collection was implemented. AEs are presented by MedDRA (v.23.0) preferred term (PT).

Continuous variables are presented as median (interquartile range (IQR)) since, according to the Shapiro–Wilk test, they followed a non-normal distribution. Median PFS, TTR, and DoR were estimated using the Kaplan–Meier method. The Greenwood’s formula was used for the standard error of survival estimates. PFS was calculated as the time from POM/LoDex initiation to the date of confirmed PD or death due to any cause, whichever occurred first. Documented PD in patients who died within 48 days after the PD was considered as a confirmed event. Patients without PD or death, or who had not started new anti-myeloma therapy until the end of the observation period were censored on the date of their last disease assessment documenting the absence of PD, while patients who started a new anti-myeloma therapy before the documentation of PD were censored on the date of their last disease assessment on or before the start date of the new anti-myeloma therapy. TTR was calculated as the time from POM/LoDex initiation to the first documented response (≥PR) and DoR as the time from the first confirmed documented response to PD or death due to any cause. The log-rank test was used to compare the survival distribution between groups of patients. 

The association of factors of interest with time-to-event effectiveness outcomes was examined using univariable and multivariable Cox regression analyses. The proportionality of hazards assumption was evaluated using the proportional hazards (PH) statement in Statistical Analysis System (SAS). Only the best fitted multivariable models selected on the basis of the minimization of Akaike information criterion are presented. The following factors were included in the initial stage of the stepwise procedures: age at MM diagnosis (65 years cut-off), Eastern Cooperative Oncology Group (ECOG) performance status at baseline, treatment line of POM/LoDex, baseline serum lactate dehydrogenase (LDH), presence of at least one comorbidity, POM/LoDex treatment initiation after clinical relapse, refractoriness to bortezomib and/or LEN, bortezomib in the immediately prior treatment line, LEN in the immediately prior treatment line, sex, and time from initial diagnosis to POM/LoDex initiation. Factors with a missing rate exceeding 10% and factors with zero number in at least 1 group were excluded from the initial step of the stepwise procedure. Firth’s penalized likelihood approach of logistic regression was used in order to account for separability issues.

Statistical analyses were performed using SAS statistical analysis software (version 9.4) (SAS Institute, Cary, NC, USA). All statistical tests were two-sided and were performed at a 0.05 significance level.

### 2.4. Sample Size Determination

Considering that the median PFS would be about 5.4 months compared to the lowest of 4.0 months shown in the clinical trial setting [16,19], in which the majority of patients were double refractory and heavily pretreated, and assuming a 38-month accrual period and a minimum 12-month observation period, we found that the assessment of 91 subjects was required in order to reject a median PFS estimate of 4.0 months (with lower and upper critical values of 3.3 and 5.0 months, respectively) against a median PFS of 5.4 months in our study population, with power 80% (specifically 80.2%), significance level a = 0.05, and a two-tailed test. Therefore, in order to account for a non-evaluable/drop-out rate of about 10%, we considered 100 patients to be adequate in order to ensure the aforementioned sample size for the final statistical analysis.

## 3. Results

### 3.1. Patients

**Figure 1:**
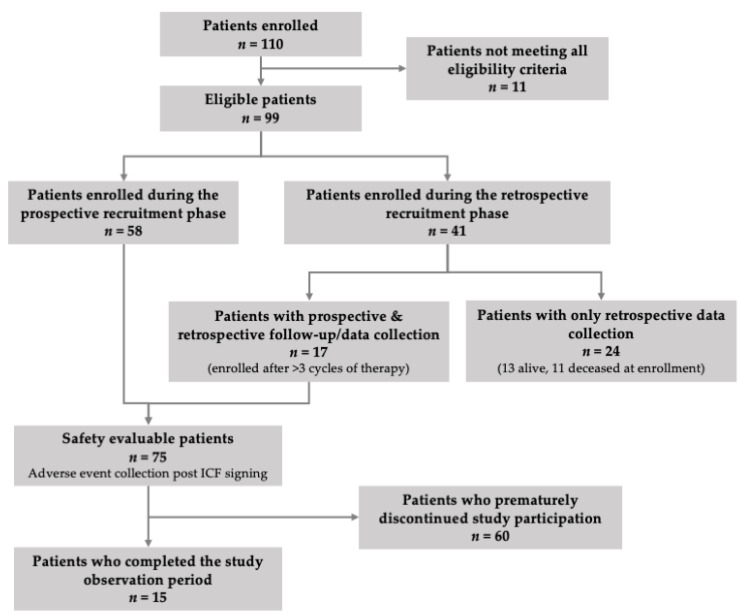
Patient disposition in the study.

Between 16 November 2017 and 21 February 2019, 99 eligible patients were enrolled in the study by 18 hematology specialists practicing in private/public hospitals in Greece. Of the eligible population, 58.6% (58/99) were enrolled during the prospective and 41.4% (41/99) during the retrospective recruitment phase (Figure 1). The median (IQR) study observation period of the overall eligible study population was 8.8 (4.2–15.4) months. 

The patients’ demographic and baseline characteristics are shown in Table 1. The patients’ median age at baseline was 71.8 years, with 78.8% being >65 years old. At baseline, 40.4% of the patients had only relapsed disease, 8.1% had only refractory disease, and 51.5% of the patients had both relapsed and refractory MM. In accordance with the eligibility criteria, all patients had received prior treatment with LEN and bortezomib, with 48.5% reported to be refractory to LEN, 41.4% to bortezomib, and 33.3% to both (Table 1). Immediately prior to POM/LoDex initiation, 96.0% of the patients had received steroids, 66.7% IMiDs (LEN in 64.6%), 42.4% PIs (bortezomib in 32.3%), 25.3% chemotherapy agents, 12.1% daratumumab, and 4.0% other targeted therapies (panobinostat and selinexor).

### 3.2. Treatment Characteristics

Among eligible patients, 50.5% were initiated on POM/LoDex treatment in the third treatment line (Table 1). POM was initiated at 4 mg/day on days 1–21 of a 28-day cycle in 75.8% of the patients. In 50.5% of them, POM was initiated with the recommended dexamethasone dosing schedule of 40 mg on days 1, 8, 15, and 22 of a 28-day cycle. The most common reasons for not receiving the recommended starting POM dosing schedule were patient’s age (in 9/24 (37.5%) of the patients), hematologic conditions (anemia, thrombocytopenia, neutropenia; in 7/24 (29.2%)), and renal impairment (in 6/24 (25.0%)).

**Figure 2:**
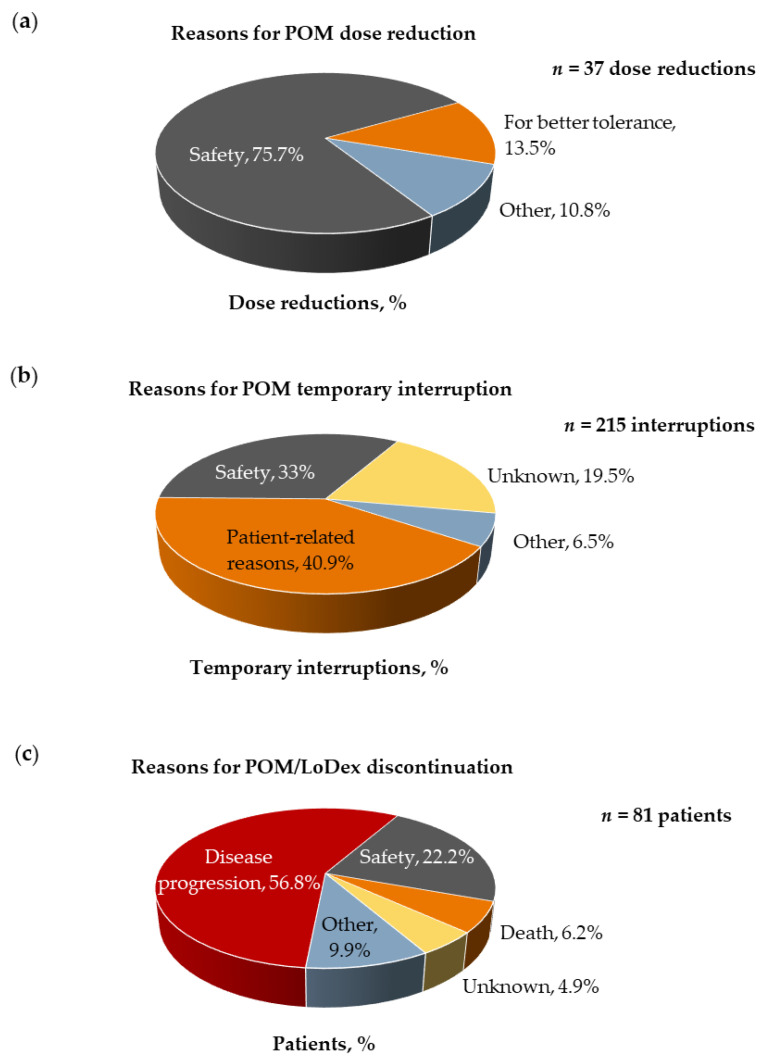
Reasons for pomalidomide (POM) treatment modifications and permanent discontinuation. (**a**) Reasons for POM dose reductions; (**b**) reasons for POM temporary interruptions; (**c**) distribution of patients who permanently discontinued POM/low-dose dexamethasone (LoDex) by reason for discontinuation.

A median of eight (range: 1–38) cycles of treatment were received at a median POM dose of 4 mg/day (range: 1–4), during a median of 8.3 (range: 0.3–47.6) months. The continuation rates of POM/LoDex treatment beyond 3, 6, and 12 months were 78.8%, 61.6%, and 37.4%, respectively. Overall, 68.7% of the patients underwent POM/LoDex dose reductions and/or temporary interruptions, with a total of 37 POM dose reductions and 215 POM temporary interruptions recorded for 28.3% and 59.6% of the patients, respectively. The reasons for dose reductions and drug interruptions are displayed in Figure 2a,b. In addition, 81.8% of the patients permanently discontinued POM/LoDex treatment, due to disease progression (56.8%), safety reasons/concerns (22.2%), death (6.2%), or other reasons (9.9%), including patient’s wish, refractory disease, minimal residual disease negativity, old age/poor performance status, physician’s decision due to stable disease, and plan for ASCT, whereas the reason was unknown for 4.9% of the patients (Figure 2c). 

Additional antimyeloma agent(s) were administered to 31.3% (31/99) of the patients concomitantly with their treatment with POM/LoDex, including cyclophosphamide, bortezomib, daratumumab, and carfilzomib. Furthermore, during POM/LoDex treatment, 81.8% (81/99) of the patients received prophylactic therapies (mainly thromboprophylaxis, antivirals, and antibacterials), and 24.2% (24/99) received therapies for the management of AEs (Table 2). 

### 3.3. Effectiveness

The investigator-assessed ORR, CBR, and DCR rates were 32.3%, 43.4%, and 64.6%, respectively, in the overall population. The best response rates were stringent complete or complete response in 7.1%, very good PR in 8.1%, PR in 17.2%, MR in 11.1%, and stable disease in 21.2%; the response was non-evaluable in 23.2%. In the response-evaluable population (*n* = 76), the ORR, CBR, and DCR rates were 42.1%, 56.6%, and 84.2%, respectively. In patients achieving a best response ≥PR, the median TTR was 3.2 (95% confidence interval (CI): 2.6–3.6) months and the DoR was 15.8 (95% CI: 11.3–not reached) months.

**Figure 3:**
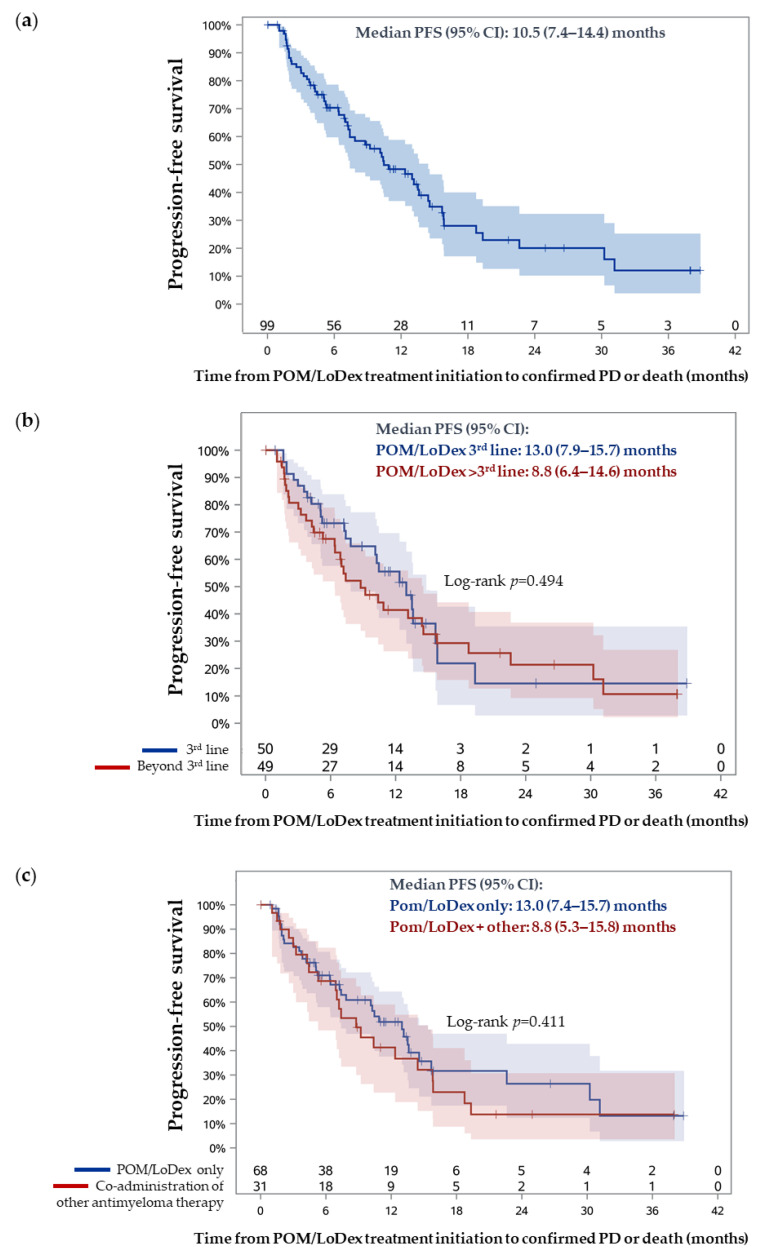
Progression-free survival (PFS) estimation by Kaplan–Meier analysis: (**a**) PFS analysis in the overall eligible population; (**b**) PFS analysis in subpopulations of patients initiated on POM/LoDex in the third versus a later line setting; (**c**) PFS analysis in subpopulations of patients receiving POM/LoDex only versus those receiving additional antimyeloma agents.

During a median follow-up period of 13.8 months (95% CI: 11.3–25.0, Kaplan–Meier estimate), the median PFS was 10.5 months (95% CI: 7.4–14.4) (Figure 3a). The Kaplan–Meier estimated that 12-, 24-, and 36-month PFS rates were 48.3%, 20.1%, and 12.0%, respectively. The median PFS was not impacted by the treatment line of POM/LoDex initiation (*p* = 0.494), or by the co-administration of other antimyeloma agents (*p* = 0.411) (Figure 3b,c). 

Patients with ECOG performance status at baseline <2 were shown to have higher odds of achieving a best response ≥PR on POM/LoDex treatment by multivariable logistic regression analysis (Table 3). Furthermore, according to multivariable Cox regression analyses, high baseline LDH levels and male sex were identified as negative predictors of PFS, while male sex was also associated with a shorter DoR. Lastly, initiation of POM/LoDex treatment after a clinical relapse and initiation of POM/LoDex in the third line treatment setting were found to be associated with a shorter TTR (Table 3). Despite being included in the initial step of the multivariable analyses, administration of LEN in the immediately prior treatment line and refractoriness to LEN and/or bortezomib were not retained in any of the final multivariable models and were not shown to be associated with any of the examined effectiveness outcomes by univariable analyses.

### 3.4. Safety

During a median safety data collection period of 7.6 months (range: 0.4–18.6), 80.0% (60/75) of the safety evaluable population experienced at least one AE irrespective of seriousness and causal relationship with POM, with a total of 218 events reported (Table 4). Among the AEs with a known outcome, 67.6% (127/188) had completely resolved at the end of the safety data collection period. A total of 73 events during the safety data collection period—comprising 40 non-hematological toxicities experienced by 30.7% (23/75) of the patients and 33 hematological toxicities experienced by 18.7% (14/75) of the patients—were assessed as being related to POM (ADRs). The only ADRs with a frequency ≥4% were neutropenia (13.3%), drug ineffective (8.0%), anemia (4.0%), and diarrhea (4.0%). A total of four infections experienced by four (4.0%) patients were reported as being related to POM. Overall, 15 patients experienced 25 AEs, which had a fatal outcome during the study period; none of these AEs were assessed to be causally related to POM.

## 4. Discussion

The present study provides real-world evidence on the effectiveness, drug utilization patterns, tolerability, and safety of POM/LoDex when administered in the third line setting or beyond in patients with RRMM who have experienced PD after LEN and bortezomib therapy.

In the context of the effectiveness outcomes of POWERFUL, the observed ORR is similar to the rates reported for the POM/LoDex arm in the phase 3 MM-003 trial (31%), as well as the 32.6% ORR reported in the subsequent STRATUS MM-010 trial, which included heavily pretreated patients with RRMM [16,17]. The ORR in POWERFUL is also similar to the 32.1% rate reported in the phase 2 MM-014 trial for the cohort of patients receiving POM/LoDex immediately after LEN-based treatment failure and the 35.7% rate reported in a recent meta-analysis of pooled data derived from 16 phase 2 and 3 clinical trials [7,21]. The median DoR in POWERFUL (15.8 months) was within the range of that reported in the clinical trial setting (8.3–16.6 months) [7,15], while the TTR was somewhat longer (3.2 vs. 1.9 months) [15]. Other real-world studies performed in Poland, Italy, and the United Kingdom have reported higher ORR (39.1–52.9%) than in POWERFUL, but it should be noted that the response rates in all these studies were derived in the response-evaluable population, and, therefore, they should be compared to the 42.1% rate observed in the present study [22,23,24]. The PFS benefit in POWERFUL is also comparable to the 12.2-month PFS and the 50.2% 1-year PFS rate reported in the study of Siegel et al. in which all patients had received LEN in the last treatment line [7], but it was more pronounced than that observed in other studies, where the median PFS ranged between 4.0 and 6.5 months [16,17,25]. Although in POWERFUL the response was evaluated on the basis of investigators’ response criteria (including IMWG in most but not all cases), this factor is not expected to introduce significant variation, and differences in effectiveness outcomes are more likely attributed to differences in patient baseline characteristics and prior treatments. This notion is also supported by the regression analyses, which revealed sex, ECOG PS status, and other parameters as significantly associated with specific effectiveness outcomes. In addition, in the present study, PFS was not impacted by the co-administration of other antimyeloma agents. This lack of any association may be attributed to differences in baseline characteristics between the treatment groups. Moreover, it should be noted that the analysis of PFS in patients who were receiving other concomitant antimyeloma agents included both patients who were given an additional antimyeloma agent from the start of the treatment line, as well as those in whom an antimyeloma agent was added to POM/LoDex during the course of therapy. Therefore, the lack of a difference in PFS in patients treated with Pom/LoDex doublet versus other POM-based therapies should be interpreted with caution, taking also into consideration that in order to draw conclusions about differences in treatments, it is best to perform head-to-head comparisons in patient subpopulations matched for critical baseline characteristics.

Consistent with prior studies [16,17], POWERFUL demonstrates that patients refractory to LEN (about 49% of the population) as well as those who have failed LEN in the immediately prior treatment line (about 65% of the population) can benefit from POM/LoDex treatment [7,26]. Pomalidomide retreatment after daratumumab has been also associated with clinical benefit as well [27]. The effectiveness of POM/LoDex in LEN-resistant MM observed in the clinical setting is based on its mechanism of action and is supported by evidence from experimental cell-based and animal models. Specifically, POM has been shown to be active across multiple LEN-resistant cell lines, as well as in an in vivo xenograft model of acquired resistance to LEN [28,29]. Additionally, although both POM and LEN act by binding CRBN, several pharmacological differences of the two IMiDs could explain the demonstrated ability of POM to overcome LEN resistance. For instance, in IMiD-resistant tumor cells, the levels of CRBN are reduced; however, residual CRBN levels in LEN-resistant cells have been shown to be sufficient for POM to maintain functionality [28]. Moreover, POM has higher affinity for CRBN; is twice as potent as LEN in the degradation of Ikaros [30]; and is more effective in degrading ARID2, a substrate of CRL4 CRBN [14]. Gene expression profiling in xenograft tumor cells following treatment with LEN/Dex or POM/Dex showed that they induce differential gene expression patterns, including some shared but also many unique downstream targets [29]. In sensitive cells, most genes deregulated by LEN/Dex were also deregulated by POM/Dex. However, only a minority of genes were found to be commonly deregulated in cells resistant to LEN/Dex and cells resistant to POM/Dex, indicating different mechanisms of resistance to these therapies. Interestingly, the effectiveness of POM/Dex in rescuing LEN resistance was significantly more pronounced than that of LEN/Dex in rescuing POM resistance [29]. Furthermore, we should note that approximately half of the included patients in our study were LEN-refractory and only one-third of the participants were double refractory to bortezomib and LEN. For this subgroup of patients, retreatment with LEN-based combinations could be an acceptable therapeutic option with important anticipated benefit [31]. However, there are no solid data in the literature favoring LEN retreatment over POM-based combinations. 

In addition to the importance of being able to overcome resistance to prior treatments and provide deep and durable responses, a major challenge in the management of RRMM is the tolerability of therapies by patients. Patients with MM commonly suffer from disease-related comorbidities and marked immunosuppression, along with the toxic effects of therapy. All these result in a high incidence of AEs. In the present study, during the safety data collection period, the overall AE incidence rate was 80.0%, while the POM-related AE incidence rate was 42.7%. Infections/infestations were experienced by 37.3% of the patients overall; the POM-related incidence of infections was 4.0%. In comparison, the rates of grade 3 or higher pneumonia ranged between 8% and 13% in the MM-003 and MM-010 clinical trials [16,17]. The rate of grade 3 or higher POM-related hematological toxicities was 8%, with neutropenia being the most common (4.0%), followed by anemia and thrombocytopenia (2.7% each). The observed rates of POM-related AEs are significantly lower than those reported in other studies in which they were much higher than 10%, and according to which these events are listed as very common in the product’s SmPC [18,32]. Hematological toxicities including neutropenia (up to 50%), anemia (up to 37%), and thrombocytopenia (up to 26%) were frequently reported in both the MM-003 and MM-010 clinical trials evaluating POM with dexamethasone [16,17]. Moreover, in POWERFUL, there was only a single case of thrombosis (MedDRA PT: deep vein thrombosis) related to POM, which was consistent with the rare incidence of deep vein thrombosis in the STRATUS clinical trial [17]. The relatively low frequencies of both hematologic and non-hematologic AEs reported in the present study could be directly related to the high percentage of patients receiving concomitant therapies for prophylaxis (81.8%). In particular, thromboprophylaxis was received by 56.6% of the patients, while 51.5% of the patients received antivirals, 45.5% antibacterials, and 14.1% antimycotics, while only 10.1% received filgrastim. The benefits of prophylactic therapy concomitantly with POM/LoDex is increasingly being recognized and specific recommendations have been developed [33,34,35,36]. It would be interesting to perform further studies examining the potential reasons for not administering such agents in a higher percentage of the patients in the real-world clinical practice. It should be also noted that in POWERFUL the AEs were generally well managed, except for the 22% of the overall population who permanently discontinued treatment due to AEs. In addition, 19% and 35% of the enrolled patients underwent dose reductions and temporary treatment interruptions due to AEs, while treatment was ongoing in more than one-third of the patients after 12 months. Administration of concomitant medications (e.g., antianemic preparations, filgrastim, antibiotics) for the management of AEs is likely related to the fact no POM-related hematological toxicity or infection resulted in treatment discontinuation. 

Limitations of the study regarding the effectiveness outcomes include the fact that response assessments were performed on the basis of criteria used by the investigators in their routine practice and not solely on the basis of the IMWG criteria, which limits comparability to other studies; however, it reflects routine clinical practice in the country. Furthermore, about one-quarter of the patients did not have an evaluable/known best response assessment, therefore reducing the robustness of the response rates. A high missing rate in some critical baseline parameters (e.g., presence of high-risk cytogenetics) also limited the assessment of their association with the effectiveness outcomes, resulting in their exclusion from the final multivariable models. With respect to the safety analysis, caution should be made in noting that this analysis included only prospectively collected AEs in the 75 patients comprising the safety evaluable population, and events that occurred after treatment initiation but prior to study entry might have been missed. This may further influence the association between the incidence of AEs and the use of prophylactic and concomitant treatments for the management of AEs, which are based on the overall population. On the other hand, among the strengths of this study, we should note that this study took place in a normal clinical setting under real-life conditions and, thus, it is more representative of both the study population of interest and the clinical outcomes under evaluation. Moreover, the geographic diversity of the participating sites, as well as the fact that the patients were enrolled by physicians practicing in both the public and the private sector, reflects various treatment paradigms across the country.

## 5. Conclusions

In the present real-world study conducted in Greece, POM/LoDex demonstrated a long PFS when administered to patients with RRMM and at least two prior lines of therapy. Neither the treatment line in which POM/LoDex was administered nor the receipt of concomitant antimyeloma agents had an impact on its effectiveness. POM displayed a limited and manageable safety profile with no new safety signals emerging. Concomitant prophylactic therapies likely contributed to the low POM-related AE rate. The results of the study support the validity of POM/LoDex as a therapeutic option for a broad group of patients, including patients refractory to LEN and those who have failed LEN in their last line of treatment. 

## Figures and Tables

**Table 1:** Patient demographic and baseline characteristics.

**Patient and Disease Characteristics at Baseline (*n = 99*)**	
**Males, *n* (%) (*n = 99*)**	53 (53.5)
**Age at baseline (*n = 99*)**	
Median (IQR), years	71.8 (65.8–76.6)
>65 years, *n* (%)	78 (78.8)
>75 years, *n* (%)	29 (29.3)
**Time from initial MM diagnosis to baseline [years; median (IQR)] (*n = 99*)**	**3.8 (2.3–6.6)**
**Medical/surgical history (past/ongoing), *n* (%) (*n = 99*)**	**87 (87.9)**
Presence of comorbidities, *n* (%)	74 (74.7)
Number of comorbidities [median (IQR)]	1.0 (0.0–3.0)
Presence of comorbidities related to MM or its complications, *n* (%)	35 (35.4)
**IgG/IgA MM subtype, *n* (%) (*n = 76*)**	50 (65.8)/26 (34.2)
**ISS Stage I/Stage II/Stage III disease, *n* (%) (*n = 82*)**	9 (11.0)/24 (29.3)/49 (59.8)
**High-risk cytogenetics ^1^ at baseline or at initial diagnosis, *n* (%) (*n = 57*)**	12 (21.1)
**Relapsed and refractory/relapsed/refractory MM, *n* (%) (*n = 99*)**	51 (51.5)/40 (40.4)/8 (8.1)
**Serum LDH (*n = 89*)**	
Median (IQR), U/L	176 (145–242)
>ULN, *n* (%)	20 (22.5)
**Serum creatinine (*n = 71*)**	
Median (IQR), mL/min	65.6 (43.8–93.0)
<60 mL/min, *n* (%)	31 (43.7)
**ECOG PS 0/1/2/3, *n* (%) (*n = 99*)**	18 (18.2)/46 (46.5)/24 (24.2)/11 (11.1)
**POM/LoDex initiation in line 3/4/5/6, *n* (%) (*n = 99*)**	50 (50.5)/26 (26.3)/17 (17.2)/6 (6.1)
**Prior MM treatments, *n* (%) (*n = 99*)**	
**Steroids**	98 (99.0)
**IMiDs:** any/lenalidomide/thalidomide	99 (100)/99 (100)/26 (26.3)
**PIs:** any/bortezomib/carfilzomib/ixazomib	99 (100)/99 (100)/10 (10.1)/8 (8.1)
**Chemotherapy ^2^**	87 (87.9)
**mAbs**	15 (15.2)
**Other targeted therapies**	6 (6.1)
**Autologous stem cell transplantation**	24 (24.2)
**Refractoriness to prior treatments, *n* (%) (*n = 99*)**	
**To IMiDs:** any/lenalidomide/thalidomide	48 (48.5)/48 (48.5)/2 (2.0)
**To PIs:** any/bortezomib/carfilzomib/ixazomib	42 (42.4)/41 (41.4)/2 (2.0)/3 (3.0)
**Double refractory to bortezomib and lenalidomide**	33 (33.3)
**To daratumumab**	5 (5.1)
**To other agents** (including cisplatin, cyclophosphamide, dexamethasone, doxorubicin, etoposide, and melphalan)	9 (9.1)

^1^ Presence of the 17p deletion, and/or the t(14;16) and/or t(14;20) translocations. ^2^ Chemotherapy included cyclophosphamide (*n* = 69); melphalan (*n* = 32); doxorubicin (*n* = 17); etoposide (*n* = 10); cisplatin (*n* = 9); bendamustine (*n* = 2); vincristine (*n* = 2); and vinblastine (*n* = 1). ECOG PS, Eastern Cooperative Oncology Group performance status; Ig, immunoglobulin; IMiDs, immunomodulatory drugs; IQR, interquartile range; ISS, International Staging System; LDH, lactate dehydrogenase; mAbs, monoclonal antibodies; MM, multiple myeloma; POM/LoDex, pomalidomide plus low-dose dexamethasone; PIs, proteasome inhibitors; SD, standard deviation; ULN, upper limit of normal.

**Table 2:** Concomitant pharmacological therapies for the management of multiple myeloma and adverse events, as well as for prophylaxis.

**Concomitant Antimyeloma Therapies Received with POM/LoDex at Any Time, *n* (%) (*n = 99*)**		
**Any antimyeloma agent**	31 (31.3)	
Cyclophosphamide	16 (16.2)	
Bortezomib	8 (8.1)	
Daratumumab	7 (7.1)	
Carfilzomib	1 (1.0)	
**Concomitant therapies in >5.0% of the patients, *n* (%)** **(*n = 99*)**	**Prophylaxis**	**Adverse Events**
**Any concomitant therapy**	81 (81.8)	24 (24.2)
Antithrombotic agents	56 (56.6)	1 (1.0)
Antivirals for systemic use	51 (51.5)	3 (3.0)
Antibacterials for systemic use ^1^	45 (45.5)	10 (10.1)
Drugs for acid related disorders	33 (33.3)	1 (1.0)
Antimycotics for systemic use	14 (14.1)	2 (2.0)
Antigout preparations ^2^	11 (11.1)	
Immunostimulants (filgrastrim)	10 (10.1)	8 (8.1)
Antianemic preparations ^3^	5 (5.1)	3 (3.0)
Drugs for treatment of bone diseases	6 (6.1)	

^1^ Includes the combination of sulfamethoxazole with trimethoprim in 37 of the 45 patients that received antibacterials as prophylaxis. ^2^ Includes allopurinol in 10 patients and febuxostat in one patient. ^3^ Includes erythropoietin-stimulating agents (ESAs) in all five patients that received antianemic preparations as prophylaxis and in two patients that received ESAs for management of AEs; one patient received ferrous sulfate for management of AEs. AE, adverse event; POM/LoDex, pomalidomide plus low-dose dexamethasone.

**Table 3:** Multivariable logistic regression and Cox regression analyses for the association of selected factors with POM/LoDex effectiveness.

	**Parameter**	**OR**	**95% CI**	***p*-Value**
ORR (*n = 99*)	Sex (male vs. female)	0.44	0.18–1.07	0.071
	ECOG PS at baseline (<2 vs. ≥2)	4.55	1.54–13.48	**0.006**
	**Parameter**	**HR**	**95% CI**	***p*-Value**
PFS (*n = 84*)	Sex (male vs. female)	2.08	1.12–3.89	**0.021**
	POM/LoDex initiation after clinical relapse	1.79	0.94–3.42	0.076
	Bortezomib in the immediately prior line	0.55	0.28–1.06	0.075
	Baseline serum LDH (>ULN vs. ≤ULN)	2.84	1.39–5.81	**0.004**
TTR (*n = 32*)	POM/LoDex initiation after clinical relapse	3.91	1.44–10.60	**0.007**
	Line of POM/LoDex initiation (3 vs. >3)	6.65	2.14–20.63	**0.001**
	Bortezomib in the immediately prior line	0.48	0.17–1.37	0.171
DoR (*n = 32*)	Sex (male vs. female)	9.75	1.84–51.66	**0.007**
	Age at MM diagnosis (<65 vs. ≥65 years)	0.31	0.06–1.45	0.136

CI, confidence interval; DoR, duration of response; ECOG PS, Eastern Cooperative Oncology Group performance status; HR, hazard ratio; LDH, lactate dehydrogenase; MM, multiple myeloma; OR, odds ratio; ORR, overall response rate; PFS, progression-free survival; POM/LoDex, pomalidomide plus low-dose dexamethasone; TTR, time to response; ULN, upper limit of normal.

**Table 4:** Adverse events reported during the safety data collection period in the safety evaluable population.

**Incidence (*n = 75*)**	**Non-Serious**		**Serious**		**Overall**	
	***n_events_***	***n* (%)**	***n_events_***	***n* (%)**	***n_events_***	***n* (%)**
AEs	135	45 (60.0)	83	36 (48.0)	218	60 (80.0)
AEs related to POM	42	23 (30.7)	31	14 (18.7)	73	32 (42.7)
AEs leading to POM discontinuation	6	6 (8.0)	21	15 (20.0)	27	21 (28.0)
POM-related AEs leading to POM discontinuation	4	4 (5.3)	3	3 (4.0)	7	7 (9.3)
Infections/infestations	18	15 (20.0)	17	15 (20.0)	35	28 (37.3)
Infections/infestations related to POM			4	3 (4.0)	4	3 (4.0)
Thrombosis/deep vein thrombosis	2	1 (1.3)	1	1 (1.3)	3	2 (2.7)
Deep vein thrombosis related to POM			1	1 (1.3)	1	1 (1.3)
					***n_events_***	***n* (%)**
**Grade ≥3 POM-related AEs**					22	10 (13.3)
**Grade ≥3 POM-related hematological toxicities**					14	6 (8.0)
Neutropenia					3	3 (4.0)
Anemia Thrombocytopenia					4	2 (2.7)
					4	2 (2.7)
Platelet count decreased					2	1 (1.3)
Neutrophil count decreased					1	1 (1.3)
**Grade ≥3 POM-related non-hematological toxicities**					8	4 (5.3)
Acute kidney injury/renal impairment					2	2 (2.7)
Back pain					1	1 (1.3)
Deep vein thrombosis Device (catheter) related infection Diarrhea					1	1 (1.3)
					1	1 (1.3)
					1	1 (1.3)
Gastric hemorrhage					1	1 (1.3)
Renal impairment					1	1 (1.3)
Urinary tract infection					1	1 (1.3)

AE, adverse event; POM, pomalidomide.

## Data Availability

The data presented in this study are contained within the article. The data are not publicly available due to restrictions that apply to the availability of the data (e.g., privacy or ethical). Datasets from this study may be available upon request from the corresponding author and provided upon approval from the sponsor and in accordance with data privacy and ethical provisions.
